# Efficacy of methylsulfonylmethane supplementation on osteoarthritis of the knee: a randomized controlled study

**DOI:** 10.1186/1472-6882-11-50

**Published:** 2011-06-27

**Authors:** Eytan M Debbi, Gabriel Agar, Gil Fichman, Yaron Bar Ziv, Rami Kardosh, Nahum Halperin, Avi Elbaz, Yiftah Beer, Ronen Debi

**Affiliations:** 1 Department of Orthopedics, Assaf Harofeh Medical Center, Zerifin, Israel

## Abstract

**Background:**

Patients with osteoarthritis (OA) take a variety of health supplements in an attempt to reduce pain and improve function. The aim of this study was to determine the efficacy of methylsulfonylmethane (MSM) in treating patients with knee OA.

**Methods:**

This study was a prospective, randomized, double-blind, controlled clinical trial. Forty nine men and women 45-90 (mean 68 ± SD 7.3) years of age with knee OA according to the American College of Rheumatology clinical criteria for OA of the knee and with radiographic confirmed knee OA were enrolled in the study and randomly assigned into 2 groups: One received MSM in doses of 1.125 grams 3 times daily for 12 weeks and the other received a placebo in the same dosing frequency. The primary outcomes were the WOMAC Osteoarthritis Index for pain, stiffness and physical function, the Aggregated Locomotor Function (ALF) test that evaluates each patient's physical function, the SF-36 quality of life health survey and the visual-analogue-scale (VAS) for pain. The secondary outcomes were Knee Society Clinical Rating System for Knee Score (KSKS) and Function Score (KSFS). Patients were assessed at baseline, 6 weeks and 12 weeks. All continuous variables were tested by the Kolmogorov-Smirnov test for Normal distribution. Changes within the groups and differences between the groups were calculated by repeated measures of analysis (ANOVA) with one nested variable.

**Results:**

There were significant differences between treatment groups over time in WOMAC physical function (14.6 mm [CI: 4.3, 25.0]; p = 0.04) and in WOMAC total score (15.0 mm [CI: 5.1, 24.9]; p = 0.03). Treatment groups did not differ significantly in WOMAC pain (12.4 mm [CI: 0.0, 24.8]); p = 0.08) or WOMAC stiffness (27.2 mm [CI: 8.2, 46.2]; p = 0.08). There was a non-significant difference in SF-36 total score between treatment groups (11.6 [CI: 1.0, 22.1]; p = 0.54). A significant difference was found between groups in VAS for pain (0.7 s [CI: -0.9, 2.4]; p = 0.05). Secondary outcomes showed non-significant differences between the two groups.

**Conclusions:**

Patients with OA of the knee taking MSM for 12 weeks showed an improvement in pain and physical function. These improvements, however, are small and it is yet to be determined if they are of clinical significance.

**Trial Registration:**

ClinicalTrials.gov: NCT01188213

## Background

Osteoarthritis (OA) is the primary cause of disability in the elderly, affecting nearly 27 million individuals in the United States alone [1]. Knee OA is the most common type of OA, with an estimated 12.1% of adults in the United States suffering from pain and functional limitations [2]. Given the association of OA with old age, these figures can be expected to rise significantly with the aging of the baby boomer population in the United States and with the increased longevity in the world's population. Currently, there is no cure for OA and treatment is focused on reducing pain and improving function [3].

Many drugs have arisen in an attempt to alleviate pain and disability, including acetaminophen, nonsteroidal anti-inflammatory drugs (NSAIDs) such as cyclooxygenase-2 (COX-2) inhibitors, and others [4,5]. Knee OA patients have also turned to surgical interventions and complementary and alternative medicine (CAM) as they have some ameliorating effects as well [3,6]. Due to recent safety concerns regarding COX-2 selective drugs, patients have turned to dietary supplements sold over-the-counter (OTC) that claim to be safer in the long-term treatment of OA. These include glucosamine and chondroitin sulfate, both of which have been examined in previous studies [7-9]. Methylsulfonylmethane (MSM) is another OTC drug that is often sold in combination with glucosamine and chondroitin sulfate. MSM can be bought at health food stores and on the internet in products such as creams and capsules.

MSM is an organosulfur molecule that can be synthesized commercially from dimethylsulfoxide (DMSO). MSM is even naturally present in the human body as it is metabolized from ingested DMSO. It can be found in cerebrospinal fluid and plasma at 0-25 μmol/l concentrations [10,11]. Many properties have been attributed to MSM, some of which include chemopreventive properties, anti-inflammatory activities, anti-atherosclerotic action, prostacyclin (PGI2) synthesis inhibition and free radical scavenging activity [12-14]. Nevertheless, conclusive data on the biochemical effects and mechanism of action of MSM is minimal. Even less is known about how these effects may benefit patients with OA of the knee.

A study by Usha and Naidu found that patients with knee OA treated with MSM showed a 33% pain reduction on the visual-analogue-scale (VAS) for pain (p < 0.001) [15]. A study by Kim et al. found that knee OA patients treated with MSM showed a significant decrease in the Western Ontario and McMaster University Osteoarthritis Index visual analogue scale (WOMAC) in pain and physical function impairment (p = 0.041 and 0.045, respectively) [16]. In a meta-analysis by Brien et al., the efficacy of MSM and DMSO was evaluated in reducing pain associated with knee OA [17]. Overall a significant but non-clinically significant reduction of pain on visual analogue scale of 6.34 mm (SE = 3.49 [CI: -0.49, 13.17]) was found in favor of MSM/DMSO, with an overall effect size of 1.82. This effect size was calculated by dividing the difference between the changes seen in each treatment group over time by the estimated standard error.

A review article by Brien et al. [18] discussed the many shortcomings of studies on DMSO and MSM. In terms of dosage, the MSM dosage used by Usha (500 mg 3 times daily for 12 weeks) was lower than the recommended dosage of MSM for clinical practice. The dosage used by Kim et al. (3 g twice a day for 12 weeks) was in the upper range of the appropriate therapeutic MSM dosage. Both studies had a clinically relevant time period. In addition, the study by Usha failed to report all the primary outcomes and the study by Kim et al. showed non-clinically significant results. In terms of the safety of MSM, the study by Usha reported minor gastrointestinal (GI) side effects in 5% of patients but did not state in which treatment group. The study by Kim et al. found GI problems, headaches, insomnia, fatigue and/or concentration problems in 57% of patients in the MSM group [18]. Other literature on MSM have also reported unproven side effects of increased blood pressure, increased effectiveness of anticoagulants and elevated liver function tests [19]. Based on these findings, Brien et al. suggested that future studies on MSM are needed to identify the optimum dosage and safety of MSM. They believe that future large-scale, long-term phase II studies are needed [18].

The purpose of this study was to evaluate the efficacy and safety of MSM in treating patients with OA of the knee at a dosage of 3.375 g/d for 12 weeks. This dosage falls between those used by the studies of Usha and Kim et al. Other measurement outcomes were also added to the study, such as real-time physical function measurements, in order to gain a greater understanding of any functional changes in the patient population.

## Methods

### Patient population

All patients gave written informed consent prior to entering the study. The protocol was approved by the Institutional Helsinki Committee Registry of Assaf Harofeh Medical Center in May 2005 (Helsinki Registration Number 13/05). The study is registered in the NIH clinical trial registry, protocol No. NCT01188213. The study was conducted at the Department of Orthopedics of Assaf Harofeh Medical Center in Zerifin, Israel, from September 2005 to February 2006. Eligibility was defined as symptomatic knee OA for at least 6 months according to the clinical criteria of the American College of Rheumatology (ACR) [20] and radiographic confirmed knee OA according to the Kellgren & Lawrence scale [21]. Exclusion criteria included acute septic arthritis; inflammatory arthritis; any other type of arthritis; history of knee buckling or recent knee injury; lack of physical or mental ability to perform or comply with the treatment procedure; diabetes mellitus; fibromyalgia or other chronic pain syndromes; concurrent anti-coagulant/anti-platelet drugs; arthroscopy or intra-articular injections in the previous 3 months. Patients using other arthritis therapies (CAM, etc.) and patients using NSAIDs were required to undergo a 10-day washout period before enrollment.

### Enrollment

**Figure 1:**
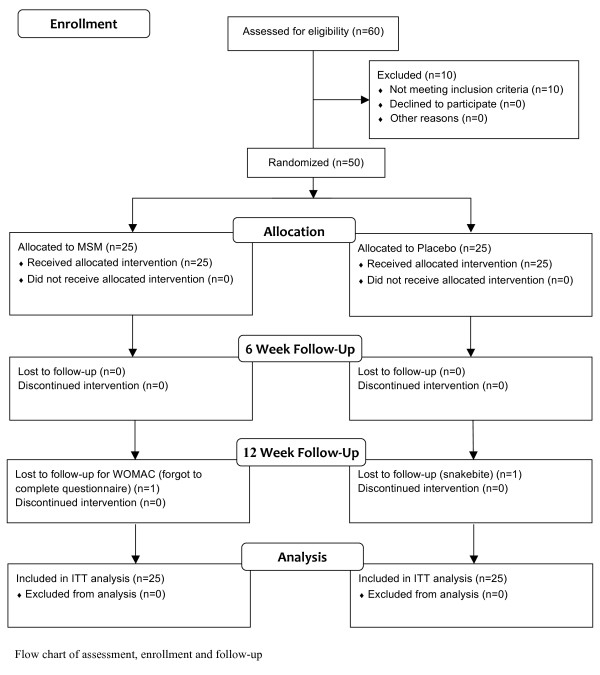
**Study flow chart**. Flow chart of assessment, enrollment and follow-up

Patients were recruited from the Assaf Harofeh Medical Center outpatient clinic. Over several months at the clinic hundreds of patients came in to be examined for regular medical problems. 60 patients who were examined by orthopedists for knee OA problems consented to enter the study. These patients were assessed by the orthopedists on staff for eligibility to the study according to the inclusion and exclusion criteria. 50 patients were determined to meet the study criteria and were immediately enrolled into the study on that day. The 50 qualified patients were assigned to an MSM (n = 25) or placebo (n = 25) group (Figure 1).

The study was a 12-week randomized, double-blind, placebo-controlled trial using random numbers (Figure 1). Random numbers were assigned as follows: Once the orthopedist on staff determined a patient's eligibility to the study, the patient was given a unique number for the duration of the study. The numbers were assigned according to the order in which they were enrolled in the study (i.e., the first patient to consent to the study and meet the study criteria was assigned the number 1). After being assigned a number and after being scored in all the study measurements for baseline data, the patient was then given a bottle of pills. All even numbers received bottles from one box and all odd numbers received bottles from the other box. The boxes were two large white cardboard boxes filled with 40 plastic bottles containing the appropriate 12-week dosage of either MSM or placebo. They were prepared by individuals who knew nothing about the study. The dosage bottles were labeled only with a serial number. The serial numbers were examined at the end of the 12 weeks and showed that the even numbered patients had received MSM and the odd numbered patients had received the placebo.

### Dosage

A dosage of 1.125 g 3x/day was chosen for the study. Purified MSM (MSM Relief, Taam Teva Altman, Israel) in 1.125 g tablets was used. Study participants were instructed to take the doses with food and to avoid taking them before bed. The placebo was comparable in all characteristics to the MSM.

### Patient evaluation

The patients were scored at baseline, 6 weeks and 12 weeks at the Assaf Harofeh Medical Center outpatient clinic. In patients suffering from bilateral knee OA, the more symptomatic knee during the initial examination was chosen for efficacy evaluation. The primary outcomes of the study were the WOMAC questionnaire [22,23], the Aggregated Locomotor Function (ALF) score [24], the SF-36 health survey score [25] and the VAS for pain [26]. The WOMAC includes pain, stiffness, physical function and aggregated total symptoms subscales that were scored from 0 mm to 100 mm (0 = no pain, 100 = worst pain). A Hebrew version of the WOMAC was used in the study, which was shown by Wigler et al. to be a reliable and valid instrument for evaluating the severity of knee OA in Israeli patients [27]. The ALF score is a sum of the mean timed scores (seconds) on three locomotor functions: time taken to walk 8 meters, time taken to ascend and descend 7 stairs, and time taken to transfer from a sitting to standing position. The SF-36 is a health survey on pain and quality of life that is scored from 0-100 (0 = worst pain and quality of life, 100 = no pain and the best quality of life). The VAS is a subjective measurement that the patient reports on a 10 cm horizontal line, where 0 indicates no pain and 10 the worst pain. The VAS is particularly useful in assessing changes in pain for individuals receiving therapy [28].

Secondary outcomes were the Knee Society Clinical Rating System [29,30] scores. This system is split into two parts: the Knee Society Knee Score (KSKS) and the Knee Society Function Score (KSFS). These are both graded from 0-100 points, with 100 being the best possible quality of the knee joint or the best functional use of the knee joint, respectively.

At each follow-up patient compliance and safety was evaluated. For compliance, patients were asked directly if they were taking their medication as they were instructed. The number of pills left in the pill bottle was also counted at each follow-up. For safety, patients were asked if there were any side effects or symptoms they were experiencing that they hadn't experienced before the study. Each patient ended the study on the day he or she came in for the final follow-up. The entire study ended on the day that last patient came in for the final follow-up in February 2006. There were no differences in follow-up time between patients.

### Statistical analysis

The criteria for a clinical response to a treatment have been defined by the Outcome Measures in Rheumatology Clinical Trials (OMERACT) and Osteoarthritis Research Society International (OARSI). These definitions are specific to visual-analogue-scales graded from 0-100 mm that can stand-alone or as part of a larger scale such as the WOMAC questionnaire. A clinical response is considered either an improvement in pain or in function of at least 50% with a decrease of 20 mm on the VAS for pain or function, or an improvement in both pain and function of at least 20% with a decrease of 10 mm on the visual-analogue-scales.

We examined the randomized assignment process by testing the hypothesis that the two groups were comparable in baseline scores of all outcomes as well as age and BMI. Chi-Square tests were used for comparing gender and BMI between the two groups. Intention to treat (ITT) analysis was carried out in order to include all the randomized patients within the analysis. In the case of any study drop-outs, the same values that were measured in the follow-up before drop-out were used for the follow-ups after drop-out.

All continuous variables were tested by the Kolmogorov-Smirnov test for Normal distribution. Changes within the groups and differences between the groups in primary and secondary outcomes were calculated by repeated measures analysis (ANOVA) with one nested variable. The one nested variable was made up of the two study groups: MSM and placebo. Additional nested variables were added to the analysis in order to adjust for any baseline differences in the distribution of certain variables (e.g. gender) between the groups. Results before and after adjustment are presented. The results from the ITT analysis were also adjusted for any baseline differences between groups.

We examined three tests of significance: difference in changes over time between groups, total changes over time for both groups and difference between groups in general. These three tests are appropriate for examining our hypothesis, which assumes: a) clinically significant improvement in scale for the active group; b) no clinically significant improvement in scale for the control group; c) no advantage in scale for the active group compared to the control group at baseline. The repeated measures analysis takes into account all three tests and a multiple comparisons correction. The analysis was performed by an external statistician using SPSS software version 14.0 (SPSS, Chicago).

## Results

There were significant differences between the groups at baseline in gender, BMI and ALF scores (Table 1). The analyses performed on the data and the results found were adjusted to account for these differences. Tables 2 and 3 present the unadjusted and adjusted results both after 6 weeks and at the 12-week endpoint. On the final follow-up, 1 patient from the placebo group dropped out due to a snakebite and 1 patient from the MSM group forgot to complete the WOMAC questionnaire (Figure 1). These patients were still included within the ITT analysis. All patients in each group reported full compliance with the treatment protocol when questioned at each follow-up. In addition, pill bottles had the appropriate number of pills at each follow-up. None of the patients reported any side effects or symptoms that they hadn't experienced before the study.

**Table 1:** Baseline characteristics of the two study groups

Characteristic	MSM (N = 25)	Placebo (N = 25)	p value*
Kellgren and Lawrence Grade			
Grade 1	4	8	
Grade 2	7	6	
Grade 3	4	6	
Grade 4	10	5	0.324
Male gender	4	13	0.007*
Age	67.0 ± 9.8	71.0 ± 8.3	0.203
Body mass index (BMI)	31.4 ± 5.4	28.6 ± 3.09	0.037*
WOMAC (0-100 mm scale)			
Pain subscale	42.4 ± 25.7	45.9 ± 27.4	0.641
Stiffness subscale	46.7 ± 24.8	43.0 ± 35.6	0.671
Function subscale	39.9 ± 21.2	47.4 ± 21.9	0.221
Aggregated total score	40.9 ± 20.3	47.0 ± 22.2	0.100
ALF (seconds)			
8 meter walk time	9.4 ± 3.9	8.2 ± 2.1	0.172
Stand-sit-stand transfer time	10.4 ± 5.3	8.4 ± 2.4	0.106
Stair ascent and descent time	23.9 ± 15.1	16.5 ± 5.8	0.029*
ALF score	43.6 ± 23.2	33.1 ± 9.6	0.043*
SF-36 (0-100 scale)			
SF-36 score results	54.1 ± 18.9	58.0 ± 17.2	0.446
Visual-analogue-scale for pain	3.78 ± 3.04	4.60 ± 2.74	0.320
Knee Society Knee Score	60.04 ± 15.6	59.5 ± 17.1	0.911
Knee Society Function Score	60.4 ± 17.2	58.9 ± 13.1	0.734

Values are presented as mean ± SD.

* The significance threshold was set at p ≤ 0.05.

MSM = methylsulfonylmethane; WOMAC = Western Ontario and McMaster Osteoarthritis Index; ALF = aggregated locomotor function; SF-36 = 36-item short-form health survey.

There were significant differences between the groups at baseline in gender, BMI and ALF score. Results of the study were adjusted to account for these differences.

**Table 2:** Primary outcomes over the 12-week treatment period

	MSM (n = 25)			Placebo (n = 25)				Significance for between group differences at follow-ups	
	
	6 weeks	12 weeks	Difference 0-12 wk [CI]	6 weeks	12 weeks	Difference 0-12 wk [CI]	Between group difference [CI]	0-6 wks	0-12 wks
WOMAC									
Pain	35.5 ± 26.1	34.0 ± 24.5	-9.0 ± 24.0 [-18.9, 0.9]	47.1 ± 26.6	49.4 ± 20.8	3.5 ± 19.3 [-4.5, 11.5]	12.4 [0.0, 24.8]	0.20	0.05*
Stiffness	39.2 ± 31.0	36.0 ± 26.2	-11.7 ± 30.7 [-24.3, 1.0]	52.9 ± 30.4	58.5 ± 24.2	15.5 ± 35.8 [0.7, 30.3]	27.2 [8.2, 46.2]	0.03*	0.01*
Function	36.6 ± 23.7	33.1 ± 23.1	-7.7 ± 19.3 [-15.8, 0.3]	46.2 ± 25.1	54.3 ± 21.1	6.9 ± 17.0 [0.1, 13.9]	14.6 [4.3, 25.0]	0.51	0.01*
Total	36.6 ± 23.9	33.3 ± 22.5	-8.4 ± 17.8 [-15.8, -1.1]	47.2 ± 24.5	53.5 ± 20.3	6.5 ± 17.0 [0.5, 13.5]	15.0 [5.1, 24.9]	0.26	0.00*
ALF	36.5 ± 16.6	36.9 ± 20.7	-6.8 ± 10.3 [-11.0, -2.5]	32.2 ± 10.6	33.9 ± 10.9	0.8 ± 4.9 [-1.2, 2.8]	7.6 [2.9, 12.2]	0.02*	0.00*
SF-36	59.8 ± 19.7	62.2 ± 20.3	8.1 ± 21.8 [-0.8, 17.1]	61.1 ± 16.2	54.5 ± 15.4	-3.4 ± 14.6 [-9.5, 2.6]	-11.6 [-22.1, -1.0]	0.52	0.03*
VAS	3.30 ± 2.8	3.61 ± 2.9	-0.2 ± 3.2 [-1.5, 1.1]	5.22 ± 2.9	5.16 ± 2.22	0.6 ± 2.7 [-0.6, 1.7]	0.7 [-0.9, 2.4]	0.18	0.38

Values are presented as mean ± SD.

* The significance threshold was set at p ≤ 0.05. Intention to treat analysis was carried out with the last observation carried-forward method.

CI = 95% confidence interval; MSM = methylsulfonylmethane; WOMAC = Western Ontario and McMaster Osteoarthritis Index graded from 0-100 mm, with 100 mm being the worst symptoms; ALF = Aggregated locomotor function in seconds; SF-36 = 36-item short-form health survey graded from 0-100, with 0 being the worst pain and quality of life; VAS = Visual-analogue-scale graded from 0-10 cm for pain, with 10 cm being the worst pain.

**Table 3:** Adjusted primary and secondary outcomes over the 12-week treatment period

		Significance for between group differences at follow-ups *	
**Outcome**		**0-6 wks**	**0-12 wks**

Primary	WOMAC		
	Pain	0.43	0.08
	Stiffness	0.23	0.08
	Function	0.85	0.04*
	Total	0.60	0.03*
	ALF	0.31	0.09
	SF-36	0.98	0.54
	VAS	0.06	0.05*
Secondary	KSKS	0.43	0.09
	KSFS	0.29	0.63

* The significance threshold was set at p ≤ 0.05.

WOMAC = Western Ontario and McMaster Osteoarthritis Index; ALF = Aggregated locomotor function; SF-36 = 36-item short-form health survey; VAS = Visual-analogue-scale for pain; KSKS = Knee society knee score; KSFS = Knee society function score.

For the primary outcomes there was a significant difference between the changes in the WOMAC physical function and aggregated total symptoms in the experimental group compared to the control group after 12 weeks (p-value = 0.04 and 0.03, respectively). From baseline to the 12-week endpoint, physical function decreased by 17% (7.7 mm [CI: -0.3, 15.8]) in the MSM group and increased by 15% (6.9 mm [CI: 0.1, 13.9]) in the placebo group, corresponding to a difference of 14.6 mm [CI: 4.3, 25.0] between groups (p = 0.04). Total symptoms decreased by 20% (8.4 mm [CI: 1.1, 15.8]) in the MSM group and increased by 14% (6.6 mm [CI: 0.5, 13.5]) in the placebo group, corresponding to a difference of 15.0 mm [CI: 5.1, 24.9] between groups (p = 0.03). No significant difference was found between treatment groups over time in the WOMAC pain or stiffness subscales. Pain decreased by 21% (9.0 mm [CI: -0.9, 18.9]) in the MSM group and increased by 9% (3.5 mm [CI: -4.5, 11.5]) in the placebo group. The difference in pain improvement was 12.4 mm [CI: 0.0, 24.8] between the groups (p = 0.08). Stiffness decreased by 26% (11.7 mm [CI: -1.0, 24.3]) in the MSM group and increased by 37% (15.5 mm [CI: 0.7, 30.3]) in the placebo group, corresponding to a difference of 27.2 mm [CI: 8.2, 46.2] between groups (p = 0.08).

There was a non-significant improvement in ALF and SF-36 scores. In ALF the MSM group showed a decrease of 15% (6.8 seconds [CI: 2.5, 11.0]) in function tests. On the other hand there was only an increase of 1% (0.8 seconds [CI: -1.2, 2.8]) in the placebo group, corresponding to a difference of 7.6 seconds [CI: 2.9, 12.2] between groups (p = 0.09). In SF-36 the MSM group showed an improvement of 15% (8.1 [CI: -0.8, 17.1]) in pain and quality of life, while the placebo group instead showed a decrease of 6% (3.4 [CI: -2.6, 9.5]). This corresponded to a difference of 11.6 [CI: 1.0, 22.1] between the changes in each treatment group (p = 0.54).

The MSM group showed a significant reduction in pain on the VAS. There was a reduction of 6% (0.2 cm [CI: -1.1, 1.5]) on the VAS in the intervention group, while there was an increase of 12% (0.6 cm [CI: -0.6, 1.7]) on the VAS in the placebo group. This corresponded to a 0.7 cm [CI: -0.9, 2.4] difference between the changes in each group (p = 0.05).

The secondary outcomes are presented in Table 3. The KSKS showed a non-significant improvement of 9% (5.4 [CI: -0.1, 10.9]) in the MSM group and a reduction of 6% (3.6 [CI: -4.8, 12.1]) in the control group, corresponding to a difference of 9.0 [CI: -0.8, 18.8] between groups (p = 0.09). The KSKF showed a reduction in function in both group. The MSM group showed a decrease of 3% (2.0 [CI: -4.3, 8.3]) and the placebo group showed a decrease of 4% (2.1 [CI: -3.0, 7.3]), corresponding to a difference of 0.1 [CI: -7.8, 8.1] between groups (p = 0.63).

## Discussion

The preset study found that patients with OA of the knee treated with 3.375 g/d MSM for 12 weeks show a significant improvement in the function and total score scales of the WOMAC and in the VAS for pain compared to a placebo-controlled group. Non-significant improvements were found in the pain and stiffness scales of the WOMAC, as well as in the SF-36, ALF and KSKS.

The critical primary outcomes of the study were the pain scale of the WOMAC and VAS for pain. Kim et al. showed a 25.1% reduction in WOMAC pain in knee OA patients treated with higher MSM doses than the present study. A study by Usha showed a 33% reduction in VAS for pain in knee OA patients treated with lower MSM doses than the present study. Our results showed a mean arthritic pain decrease of just 21.1% on the WOMAC that had no significance and a decrease of 5.5% of the VAS pain scale that was significant. Neither of these, nor the results of previous studies are considered to be clinical improvements according to the criteria set forth by the OMERACT and OARSI. This falls in agreement with the meta-analysis for DMSO/MSM performed by Brien et al. that also showed a significant but non-clinically significant improvement in WOMAC or VAS scales for pain in the MSM group. Based on our calculations there was an average decrease in pain of 39.5% across the studies used in the meta-analysis. As reported by Brien et al., however, the average difference between the changes found in each treatment group was only 6.34 mm [17].

The other primary outcomes of the study were the other WOMAC scales, SF-36 and ALF. The results of the other WOMAC scales are in partial agreement with the study of Kim et al. The present study found a significant improvement in function and total score. Kim et al. found a significant improvement in pain and physical function but not in stiffness and aggregated total symptoms. Specifically, MSM improved pain by 14.6 mm (p < 0.05), physical function by 15.7 mm (p < 0.05), stiffness by 10.1 mm (p = 0.320) and total WOMAC score by 13.4 mm (p = 0.054) compared to the placebo [16].

The SF-36 results are also in agreement with the study of Kim et al. In the present study the MSM group showed a non-significant improvement of 15% in overall SF-36, compared to a worsening of symptoms of 6% in the control group. This corresponded to a score difference of 11.6 between groups. The analysis carried out by Kim et al. showed no significant differences between treatment groups in almost all SF-36 scores. A significant difference between groups was only found in the category of role limitation due to physical, with a mean change of 16.45 over 12 weeks (p = 0.021) [16].

The ALF and KSKS results showed similarities to the outcomes of the study by Usha. Our ALF results showed a non-significant 6.9-second improvement in physical function in the MSM group compared to the placebo group and the KSKS showed a non-significant 9% improvement in joint function corresponding to a 9.0-point difference between the treatment groups. Similarly the study by Usha found significant improvements in joint mobility, swelling, global evaluation and walking time [15].

All but one study outcome (i.e. KSKF) showed a greater improvement in knee OA symptoms in the MSM compared to the control, yet many of these where not significant at the end of the study. This may have occurred for several reasons. First is that there were significant baseline differences in gender, BMI and ALF scores between the groups at baseline. Almost all outcomes showed significance before adjusting for these differences (Table 2). The reasons for the baseline differences may be due to our randomization technique. We used unlabeled bottles of pills and randomized according to the order in which patients arrived at the clinic. A better randomization technique would have been to use a randomization computer model. The small sample size may also be a reason for the baseline differences.

The short time frame of the study may also have been a difficulty. It is clear from Tables 2 and 3 that all outcomes aside from KSFS continue to improve towards significance at 12 weeks. There is a good chance that had the study been continued for longer more of the results would have been significant. Nevertheless, even had many of the result been significant, it seems that most of the improvement of symptoms seen in the MSM group were minor and are likely not to have been of clinical significance. Another fault of the study is the use of a single enrollment site. This limited much of our patient pool to a local area, and limits our ability to generalize the results on a larger scale. Future studies on MSM should randomize the patient pool using computerized models, lengthen the intervention period and sample patients from different clinics and even different countries.

With regard to safety analysis, no adverse events or side effects were recorded. In contrast, very minor side effects were recorded in the study of Kim et al. These included bloating, constipation, indigestion, fatigue, inability to concentrate, insomnia and headaches. These side effects, however, were observed in equal frequency in the control group as well [16]. The lack of clinical side effects in the present study was surprising. One reason for the lack of adverse effects may be due to a difference in dosage. The dosage used by Kim et al. was almost double the amount used in the present study. Another possibility is the present study was not sensitive enough at picking up these effects. It is possible that by using more sensitive methods of evaluating side effects, such as examining medical records, liver function tests and implementing questionnaires, we would have found slight side effects in our study population. Future studies should examine the adverse effects of MSM more thoroughly.

Compared to the efficacy of standard analgesics, the efficacy of MSM is relatively smaller. For example, celecoxib and acetaminophen decreased the WOMAC pain scale by 15.5 mm and 12.5 mm [31], respectively, while MSM decreased WOMAC pain by just 9.1 mm and VAS for pain by 1.7 mm in our study. The studies by Usha and Kim et al. also showed just minor improvements relative to NSAIDs [15,16]. A meta-analysis on NSAIDs for knee OA showed them to have an average effect size of approximately 0.32 [32]. The effect size of MSM in the present study (0.28) falls slightly below this number. Compared to topical NSAIDs, which have been shown to have an effect size of approximately 0.4 [33], MSM also had a relatively smaller effect size in the present study. On the other hand, MSM had a relatively greater efficacy than intra-articular hyaluronic acid injections, which have been shown to decrease pain by only 4.3 mm [34]. These findings suggest that the efficacy of MSM is modest in comparison to the standard care for knee pain and falls below the efficacies of most standard therapies. These comparisons, however, should be interpreted with care considering that these studies did not have identical methodologies.

On the other hand, the results of this study suggest that long-term trials on MSM may yield additional and greater improvements in knee OA symptoms. There are also many serious risks associate with OA drugs, such as those recently shown for COX-2 inhibitor drugs [35,36]. MSM appears to be a relatively safe health supplement. Current research suggests that if side effects to MSM exist, they are minimal and include minor GI symptoms, headaches, insomnia and difficulty concentrating [16]. With MSM's lack of significant adverse effects and with significant, albeit minor, improvements in pain and function, patients with knee OA may benefit from adding MSM to their treatment regiment. Such a drug may be used in combination with current drugs and therapies for arthritis pain and especially if other drugs are contraindicated.

## Conclusions

Patients with OA of the knee taking MSM for 12 weeks showed an improvement in pain and physical function. The results suggest that larger and long-term studies may find additional and greater improvements in knee OA symptoms. These improvements, however, are small and it is yet to be determined if they are of clinical significance. Further trials on MSM are recommended to define the safety, efficacy and appropriate dosage of MSM. We recommend incorporating longer intervention periods, larger and wider patient populations, dose-response trials and comparisons with other health supplements and standard conventional treatments.

## Competing interests

The authors declare that they have no competing interests. The MSM and placebo used in the study were donated by the manufacturer Taam Teva. Taam Teva had no other involvement in the study.

## Authors' contributions

RD, GA and NH participated in the study conception, recruitment of patients and manuscript revisions. YBZ, RK, AE and YB participated in the recruitment of participants, implementation of treatment and data collection. ED and GF participated in the implementation of treatment, data collection, interpretation of results and drafting of the manuscript. All authors read and approved the final manuscript.

## Pre-publication history

The pre-publication history for this paper can be accessed here:

http://www.biomedcentral.com/1472-6882/11/50/prepub
